# A comparative analysis of the efficacy of anterior cruciate ligament reconstruction with autologous ligament grafting at different time points

**DOI:** 10.1002/jcla.23543

**Published:** 2020-08-26

**Authors:** Bo Hu, Feng Gao, Chunbao Li, Boqing Zhang, Mingyang An, Ming Lu, Yufeng Liu, Yujie Liu

**Affiliations:** ^1^ Medical School of Chinese PLA Beijing China; ^2^ Beijing Chaoyang Integrative Medicine Emergency Medical Center Beijing China; ^3^ Department of Sports Injury and Arthroscopy Surgery National Institute of Sports Medicine Beijing China

**Keywords:** ACL injury, knee joint function, operation timing, QOL, reconstruction with autologous ligament grafting, stability

## Abstract

**Background:**

This study was performed to compare the clinical efficacies of anterior cruciate ligament (ACL) reconstruction with autologous ligament grafting at different time points.

**Methods:**

Eighty‐five patients with ACL were categorized into two groups: Group A (GA, n = 45), who underwent early‐stage (≤3 weeks) surgery, and Group B (GB, n = 40), who underwent advanced‐stage (>3 weeks) surgery. Perioperative conditions, knee joint functions, activity and stability before and at 6 months postoperatively, changes in quality of life (QOL), good and excellent rates of knee joint functions, and incidence of complications were compared between the two groups.

**Results:**

In both groups, there was an increase in the International Knee Documentation Committee (IKDC) score, Lysholm score, and QOL and a decrease in the knee joint angle flexion limitation, angle of spread limitation, positive rates in the anterior drawer test (ADT), and Lachman test score (*P* < .05) after surgery. At 6 months postoperatively, the IKDC score, Lysholm score, and QOL were higher in GA than in GB (*P* < .05). The good and excellent rates of knee joint functions were higher in GA than in GB (93.33% vs. 77.50%) (*P* < .05).

**Conclusion:**

Anterior cruciate ligament reconstruction with autologous ligament grafting can achieve good effects whether performed in the early or advanced stage; however, the improvements in patients' knee joint functions and QOL are better in the early stage. Therefore, early ACL reconstruction with autologous ligament grafting is suggested.

## Graphical abstract

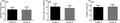

## 1 INTRODUCTION

Anterior cruciate ligament (ACL) injury is a common athletic injury involving the knee joints. The clinical manifestations of ACL include pain, edema, and limitations in joint movement/extension flexion. If the patient is not provided effective treatment in time, ACL injury may cause secondary injuries such as meniscus injury and seriously reduce the quality of life (QOL) of patients.^1^ In recent years, with the improvement of people's living standards and the popularization of various sports, the number of patients with ACL injury in China is increasing, and their QOL is seriously affected. Reconstruction with autologous ligament grafting is considered the principal treatment method for ACL injury; it can effectively restore the stability of knee joints, promote functional recovery, and improve patients' QOL.^2^, ^3^ However, there is no consensus on the optimal time to perform surgery as some researchers believe that early‐stage surgery may increase the activity of knee joints, which is conducive to functional recovery, whereas some believe that in the early stage, considering factors such as edema, blood clots, and inflammatory reactions in the joints, surgery performed at this time may increase the risks of knee joint stiffness and synarthrophysis.^4^, ^5^ Wang et al previously compared the degree of synarthrophysis and stability between patients with ACL injury who underwent early‐stage (≤3 weeks) and advanced‐stage (>3 weeks) surgery, and the results demonstrated that autologous ligament grafting at the early stage could effectively restore joint stability without increasing the degree of synarthrophysis.^6^ In this regard, we selected 3 weeks as the threshold between early‐stage and advanced‐stage surgery and retrospectively analyzed the clinical data of patients with ACL injury who underwent reconstruction with autologous ligament grafting and compared the effects of early‐stage (≤3 weeks) and advanced‐stage (>3 weeks) surgery on their knee joint functions, activity, stability, and QOL to determine the optimal time to perform ACL reconstruction with autologous ligament grafting.

## 2 MATERIAL AND METHODS

### 2.1 General materials

The clinical data of 85 patients with ACL injury who were treated at our hospital from August 2017 to February 2019 were retrospectively analyzed. Of these patients, 51 were male and 34 were female, with an average age of 25‐57 years (43.26 ± 8.33 years). Of the 85 patients, 39 were injured in sports, 29 in traffic accidents, and 17 due to other causes; 47 patients had injuries on the left side. The average follow‐up period was 6‐13 months (9.26 ± 1.36). Based on the treatment time, all 85 patients were divided into two groups: GA (n = 45), comprising those who had undergone surgery within 3 weeks (inclusive) after injury, and GB (n = 40), comprising those who had undergone surgery after 3 weeks (not inclusive) after injury.

The inclusion criteria were as follows: diagnosed with ACL injury before surgery by MRI findings, which were primarily manifested as edema thickening, signal continuity interruption, and abnormally high signal; age between 18 and 60 years; and with injuries at one knee while the other one was healthy. The exclusion criteria were as follows: surgery was performed for the knee joint at the same side; posterior cruciate ligament injury; complications of long bone injury at the same side, meniscus injury and fracture, knee joint affection, osteoarthritis, etc; and incomplete clinical data and no coordination in follow‐up investigations. This study was approved by the Ethics Committee of the Medical School of Chinese PLA. The research subjects and their families signed fully informed consent forms.

### 2.2 Method of operation

The patients were subjected to continuous epidural anesthesia. Then, they were placed on their backs and were examined for medial/lateral compartment and sulcus, fossa intercondyloidea, and bursa suprapatellaris using a conventional arthroscopic approach. The residual ends of the ACL were cleaned under arthroscopic guidance, which was removed later. A tendon graft was harvested from the same side, and a longitudinal incision was made at the medial tibia nodule to isolate, expose, and remove the tendon of the semitendinosus muscle and gracilis using a tendon taker. Muscles attached to the tendon were detached to measure the tendon length and folded diameter. The graft was then sutured for future use. With the help of a tibia localizer, the thighbone and tibia tunnels were drilled and cleaned for skeletal fragments. The ligament subject to grafting was drawn toward the bone channel, and the thighbone and tibia tunnels were fixed with interface screws. The knee joint was bent 30 times after a negative result in the anterior drawer test (ADT). The articular cavity was rinsed after confirming a satisfactory fixation, and drainage tubes were indwelled as a conventional process. Suturing was marked at the end of surgery. Surgery was performed by the same medical team for all patients. After surgery, the affected limb was dressed in pressure bandages, elevated, and secured with the help of nurses to reduce edema. For patients who experienced pain, drugs or cold compress was provided. On the first day postoperatively, patients were guided to raise their legs in a straight direction. On the third day postoperatively, they were allowed to perform out‐of‐bed activities with guidance provided that the affected limb was not loaded; patients with better performance during recovery were able to properly bend and spread their knee joint. On the seventh day postoperatively, knee joint bending exercise was performed as instructed, and at 6 weeks postoperatively, patients could walk with loads and orthoses. Training sessions were supervised to avoid violent and excessive exercise in case of secondary injury. Except for the difference in the time of surgery, the two groups were identical in other aspects.

### 2.3 Observation indices

The perioperative conditions, knee joint functions, activity and stability before and at 6 months postoperatively, changes in QOL, good and excellent rates of knee joint functions, and the incidence of complications were compared between the groups.

*Perioperative conditions:* Perioperative conditions including operation time, out‐of‐bed activity time, and length of stay (LOS) were recorded.

Knee joint functions: Knee joint functions were evaluated before and at 6 months postoperatively according to the International Knee Documentation Committee (IKDC) score^7^ and Lysholm score.^8^ The IKDC score covered physical exercises, functions, and symptoms, while the Lysholm score included lameness, climbing the stairs, crutching, and crouching. Both scores positively correlated with knee joint functions.

*Knee joint activity:* Knee joint activity was measured before and at 6 months postoperatively using a protractor, including the knee joint angle of flexion limitation and the angle of spread limitation. The average of three measurements was taken as the final.

*Knee joint stability:* Knee joint stability was evaluated by the ADT^9^ and Lachman test before and at 6 months postoperatively.^10^ In ADT, patients were instructed to lie on their back with the knee bent at 90°. The upper end of the tibia was drawn forward from the neutral position of the calf. Results were positive when the tibia nodule moved forward >5 mm. In the Lachman test, patients were instructed to lie on their back with the knee bent at 30°. Results were positive when the tibia moved forward >3 mm.

*QOL:* A QOL questionnaire was designed in accordance with the SF‐36 QOL form and the hospital's conditions to evaluate the QOL of patients before and at 6 months postoperatively. Patients were required to provide information concerning physical pain, vitality, psychological functions, and psychological health. The final score positively correlated with the QOL.

*Good and excellent rates of knee joint functions:* Patients' good and excellent rates of knee joint functions were evaluated according to the Hospital for Special Surgery knee score, including muscles, functions, pains, and flexibility. Under the centesimal system, if the score was >95 points, the result was excellent; if the score was between 80 and 95 points, it was good; if the score was between 60 and 79 points, it was acceptable; and if the score was below 60 points, it was poor.

*Complications:* Complications were evaluated and recorded, including pain at the fixation point of the absorbable bone nail, superficial infection of the tibia tunnel, and venous thrombosis in the lower extremities.

### 2.4 Statistical analysis

Statistical analysis was performed using SPSS version 18.0 software. All measurement data were tested for normal distribution. Measurement data that met the normal distribution were expressed as mean ± standard deviation. An independent t test was used to compare differences between groups, and a paired t test was used to compare differences within groups; in case of enumeration data expressed as %, comparison studies were performed using a chi‐square test. For all statistical comparisons, significance was defined as *P* < .05.

## 3 RESULTS

### 3.1 Basic data

There were no significant differences in basic data such as gender, age, and causes and places of injury as well as follow‐up period between the two groups (*P* > .05) (Table 1).

**TABLE 1:** Intergroup comparison of basic data

Group	Gender		Age (y)	Cause of injury			Place of injury		Follow‐up time (mo)
	Male	Female		Athletic injury	Traffic accident	Others	Left	Right	
GA (n = 45)	26 (57.78)	19 (42.22)	42.69 ± 5.44	20 (44.44)	16 (35.56)	9 (20.00)	24 (53.33)	21 (46.67)	9.13 ± 2.36
GB (n = 40)	25 (62.50)	15 (37.50)	43.74 ± 6.03	19 (47.50)	13 (32.50)	8 (20.00)	23 (57.50)	17 (42.50)	9.49 ± 1.92
*χ* ^2^/*t*	0.197		0.844	0.896			0.149		0.765
*P*	.657		.401	.365			.700		.446

Note

Data are expressed as n (%) or mean ± SD.

### 3.2 Perioperative conditions

**FIGURE 1:**
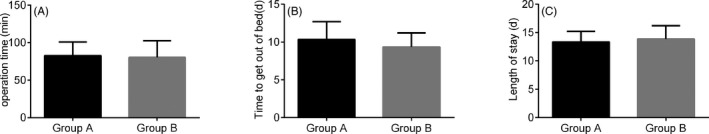
Intergroup comparison of perioperative conditions

There were no significant differences in operation time, out‐of‐bed activity time, and LOS between the two groups (*P* > .05) (Figure 1).

### 3.3 Knee joint functions

At 6 months postoperatively, the IKDC and Lysholm scores increased in both groups (*P* < .05) and were higher in GA than in GB (*P* < .05) (Table 2).

**TABLE 2:** Intergroup comparison of knee joint functions before and after surgery

Group	IKDC score				Lysholm score			
	Before the operation	At 6 mo after the operation	*t*	*P*	Before the operation	At 6 mo after the operation	*t*	*P*
GA (n = 45)	51.36 ± 5.25	92.36 ± 7.42	30.232	.000	48.36 ± 6.08	85.32 ± 8.95	22.915	.000
GB (n = 40)	52.03 ± 6.14	89.03 ± 6.33	26.536	.000	47.91 ± 7.14	81.03 ± 7.83	19.768	.000
*t*	0.542	2.210	—	—	0.314	2.338		
*P*	.589	.030	—	—	.755	.022		

Note

Data are expressed as mean ± SD.

### 3.4 Knee joint activity

At 6 months postoperatively, the knee joint angle of flexion limitation and the angle of spread limitation were reduced in both groups (*P* < .05) but exhibited no intergroup significance (*P* > .05) (Table 3).

**TABLE 3:** Intergroup comparison of knee joint activity before and after surgery (°)

Group	Angle of flexion limitation					Angle of spread limitation			
	Before the operation	At 6 mo after the operation		*t*	*P*	Before the operation	At 6 mo after the operation	*t*	*P*
GA (n = 45)	23.36 ± 2.25		13.85 ± 2.06	20.912	.000	8.36 ± 1.52	3.88 ± 1.25	14.730	.000
GB (n = 40)	23.11 ± 3.04		14.19 ± 2.32	17.946	.000	8.59 ± 1.35	4.13 ± 1.37	14.666	.000
*t*	0.434		0.716	—	—	0.734	0.853		
*P*	.665		.030	—	—	.755	.397		

Note

Data are expressed as mean ± SD.

### 3.5 Knee joint stability

The positive rates in ADT and Lachman tests were reduced in both groups 6 months postoperatively (*P* < .05) but demonstrated no significant difference (*P* > .05) (Table 4).

**TABLE 4:** Intergroup comparison of knee joint stability before and after surgery

Group	ADT test				Lachman test			
	Before the operation	At 6 mo after the operation	*χ* ^2^	*P*	Before the operation	At 6 mo after the operation	*χ* ^2^	*P*
GA (n = 45)	24 (53.33)	5 (11.11)	18.366	.000	27 (60.00)	6 (13.33)	21.101	.000
GB (n = 40)	20 (50.00)	6 (15.00)	11.168	.001	27 (67.75)	5 (12.50)	25.208	.000
*χ* ^2^	0.094	0.284	—	—	0.514	0.013	—	—
*P*	.759	.594	—	—	.473	.909	—	—

Note

Data are expressed as n (%).

### 3.6 Good and excellent rates of knee joint functions

The good and excellent rates of knee joint functions were 93.33% (42/45) in GA and 77.50% (31/40) in GB (*P* < .05) (Table 5).

**TABLE 5:** Intergroup comparison of good and excellent rate of knee joint functions

Group	Excellent	Good	Acceptable	Poor
GA (n = 45)	25 (55.56)	17 (37.78)	3 (6.67)	42 (93.33)
GB (n = 40)	20 (50.00)	11 (27.50)	9 (22.50)	31 (77.50)
*χ* ^2^	—	—	—	4.379
*P*	—	—	—	.036

Note

Data are expressed as n (%).

### 3.7 Complications

There was no statistically significant difference in the incidence of complications between the groups (*P* > .05) (Table 6).

**TABLE 6:** Intergroup comparison of the incidence of complications

Group	Pains at the fixation point of absorbable bone nail	Superficial infection of tibia tunnel	Venous thrombosis in lower extremities	Total
GA (n = 45)	0 (0.00)	1 (2.22)	1 (2.22)	2 (4.44)
GB (n = 40)	1 (2.50)	1 (2.50)	1 (2.50)	2 (7.50)
*χ* ^2^	—	—	—	0.154
*P*	—	—	—	.904

Note

Data are expressed as n (%).

### 3.8 QOL

**FIGURE 2:**
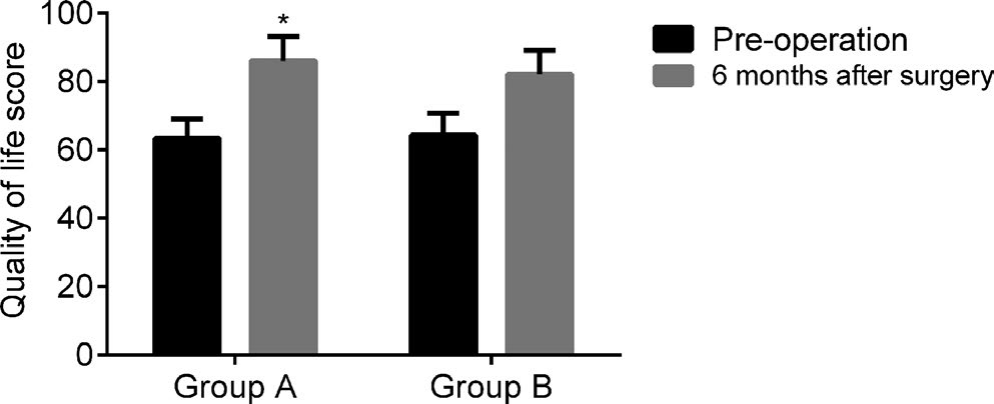
Intergroup comparison of QOL before and after surgery

The QOL score increased in both groups 6 months postoperatively (*P* < .05) and was higher in GA than in GB (*P* < .05) (Figure 2).

## 4 DISCUSSION

This study analyzed the clinical data of 85 patients with ACL injury who were treated by autologous ligament reconstruction and compared the effects of early‐stage (≤3 weeks) and advanced‐stage (>3 weeks) surgery on knee joint function, activity, stability, QOL, and other indicators. The results demonstrated that there was no significant difference between early‐stage and advanced‐stage treatment in terms of operation time, out‐of‐bed activity time, and LOS; the IKDC score, Lysholm score, and QOL increased in both groups, whereas the knee joint angle of flexion limitation, angle of spread limitation, and positive rates in ADT and Lachman tests decreased. At 6 months postoperatively, there was no significant difference in the knee joint angle of flexion limitation, angle of spread limitation, and positive rates in ADT and Lachman tests between the two groups, indicating that ACL reconstruction with autologous ligament grafting, whether performed in an early or advanced stage, can achieve good therapeutic effect and improve the activity and stability of knee joints.^11^, ^12^, ^13^, ^14^ However, some studies^15^, ^16^ reported that the effect of advanced‐stage reconstruction on the improvement of knee activity and stability was more evident. This inconsistency may be explained by the limited sample size in this study. Furthermore, at 6 months postoperatively, GA showed higher IKDC scores, Lysholm scores, and QOL and good and excellent rates of knee joint functions than GB, which was basically consistent with the findings of Zhai,^17^ signifying that early reconstruction is better than advanced reconstruction in improving knee joint functions and QOL. The reasons may be related to the following two aspects: On one hand, with early reconstruction, patients can recover to the previous competitive level as early as possible. If early reconstruction is not performed, the degeneration of the cartilage will be accelerated by the instability of the knee joint, and the risk of long‐term cartilage and meniscus injury will be increased. Early reconstruction aids in the recovery of the stability of patient's knee joint functions as soon as possible and reduces complications as reported previously.^18^, ^19^, ^20^, ^21^, ^22^ On the other hand, in early reconstruction, more ACL stumps and ligament synovium can be preserved, which is conducive to the anatomical location of the bone marrow channel and effectively promotes crawling of the reconstructed blood vessels, thereby facilitating the replacement of fibroblasts and new tissues and accelerating the healing of the ligament.^23^, ^24^ A study^25^ demonstrated that in early reconstruction, the knee joint is in an acute synovial stage and patients often experience severe pain. It is somewhat difficult to perform reconstruction surgery at this time point. In addition, early fixation of the affected limb after surgery easily increases the risk of synarthrophysis, which is not conducive to the recovery of joint functions. Therefore, reconstruction at an advanced stage is suggested. In view of the above‐described situation, it was found that in the early stage, ideal results could be achieved if patients were guided to perform exercises of muscle strength, and the knee joint swelling was eliminated before a reconstruction surgery.

In conclusion, ACL reconstruction with autologous ligament grafting could achieve good effects whether performed in the early or advanced stage, but improvements to patients' knee joint functions and QOL are better when ACL reconstruction is performed in the early stage. Therefore, early ACL reconstruction with autologous ligament grafting is suggested. However, bias could be introduced due to the limited number of samples assessed in this study and a wide range of follow‐up periods. Future studies should consider larger sample sizes and unify the follow‐up time for further exploration.

## CONFLICT OF INTEREST

None.
